# Evaluation of Indoor Air Quality Screening Strategies: A Step-Wise Approach for IAQ Screening

**DOI:** 10.3390/ijerph13121240

**Published:** 2016-12-14

**Authors:** Ling-tim Wong, Kwok-wai Mui, Tsz-wun Tsang

**Affiliations:** Department of Building Services Engineering, The Hong Kong Polytechnic University, Hong Kong 999077, China; beltw@polyu.edu.hk (L.W.); tszwun.tsang@connect.polyu.hk (T.T.)

**Keywords:** indoor air quality, assessment, screening, air-conditioned office

## Abstract

Conducting a full indoor air quality (IAQ) assessment in air-conditioned offices requires large-scale material and manpower resources. However, an IAQ index can be adopted as a handy screening tool to identify any premises (with poor IAQ) that need more comprehensive IAQ assessments to prioritize IAQ improvements. This study proposes a step-wise IAQ screening protocol to facilitate its cost-effective management among building owners and managers. The effectiveness of three IAQ indices, namely θ_1_ (with one parameter: CO_2_), θ_2_ (with two parameters: CO_2_ and respirable suspended particulates, RSP) and θ_3_ (with three parameters: CO_2_, RSP, and total volatile organic compounds, TVOC) are evaluated. Compared in a pairwise manner with respect to the minimum satisfaction levels as stated in the IAQ Certification Scheme by the Hong Kong Environmental Protection Department, the results show that a screening test with more surrogate IAQ parameters is good at identifying both lower and higher risk groups for unsatisfactory IAQ, and thus offers higher resolution. Through the sensitivity and specificity for identifying IAQ problems, the effectiveness of alternative IAQ screening methods with different monitoring parameters is also reported.

## 1. Introduction

Modern people spend over 90% of their time indoors [1]. The World Health Organization (WHO) reported 3.8 million premature deaths attributed to poor household indoor air quality (IAQ), contributing approximately 6.8% of the global mortality [2]. Growing concern about IAQ in workplaces, enclosed public places, and residential buildings have been received [3,4], which mainly focus on the potential health effects and the economic consequences of prolonged exposure to indoor air pollutants. In Hong Kong, a majority of people work in an indoor environment. Therefore, maintaining an acceptable IAQ is of utmost importance to protect the health of the general public. In view of the increasing IAQ concerns and complaints [5,6], there is an urgent need of a practical diagnostic tool for proper IAQ management.

Development of IAQ assessment tools has been proposed with two approaches: (1) health-related approach; and (2) surrogate indicator approach. Health-related IAQ assessment tools target on a dose–response relationship—also known as an exposure–response relationship—which describes the change in effect on health when exposed to a stressor over a range of exposure levels and exposure times. A successful example was reported for particulate matter 10 µm or less in diameter (PM_10_), with an increase of 0.69% in mortality for every 10 µg/m^3^ increase in PM_10_ [7]. Although PM_10_ has been proven to be closely correlated with mortality, some other IAQ parameters do not cause observable health effects unless at extremely high concentrations. Carbon dioxide (CO_2_), for example, is found to be closely associated with sick building syndrome (SBS) [8], yet its effects on health are subtle and non-lethal. As extensive research and thorough testing are required, it can be extremely expensive to develop a health-related IAQ assessment tool.

To minimize the need for and the cost of a comprehensive IAQ assessment, surrogate indicators provide an alternative approach. To assess IAQ in air-conditioned offices in Hong Kong, Hui, Wong, and Mui proposed an express assessment protocol (EAP) which uses only the top three and five contributors to unsatisfactory IAQ to identify “Excellent” and “Good” IAQ classes, respectively [9,10]. In another study, the same team used the concentration levels of three independent yet closely correlated IAQ parameters—namely CO_2_, respirable suspended particulates (RSP), and total volatile organic compounds (TVOC)—to successfully predict IAQ dissatisfaction without assessing the other nine IAQ parameters as required in the IAQ Certification Scheme by the Hong Kong Environmental Protection Department [11]. CO_2_, RSP, and TVOC were chosen because they are surrogate indicators for occupant load and ventilation rate, system filtration performance and indoor activities, and emissions from building materials and finishes, respectively.

An IAQ index is a simple and cost-effective tool for the evaluation of IAQ. This study demonstrates that using some dominant IAQ parameters for pre-assessment can identify undesirable IAQ with engineering acceptable accuracy. To facilitate cost-effective IAQ management among building owners and managers, three IAQ indices, namely θ_1_ (with one parameter: CO_2_), θ_2_ (with two parameters: CO_2_ and RSP), and θ_3_ (with three parameters: CO_2_, RSP, and TVOC) are proposed. The results are compared in a pairwise manner with respect to the minimum satisfaction levels as stated in the IAQ Certification Scheme. Through the sensitivity and specificity for identifying IAQ problems, the effectiveness of alternative IAQ screening methods with different monitoring parameters is also reported.

## 2. Materials and Methods

### 2.1. Concept of Screening Strategy

According to the threshold approach by Pauker and Kassirer [12], while no action is required for maintaining the IAQ level if the post-test probability *P′_d_* of unsatisfactory IAQ is below the testing threshold (also known as the no action threshold), immediate remediation should be given to improve the IAQ level if *P′_d_* is above the test–treatment threshold. Further tests should be performed only if *P′_d_* is between the two thresholds. In this study, the post-test failure probability *P′_d_* can be computed using Equation (1), where *O′_d_* are the post-test odds given by the pre-test odds *O_d_* and the likelihood ratio *L_r_*.

(1) $$P_{d}^{\prime }=\frac{O_{d}^{\prime }}{1+O_{d}^{\prime }};\;O_{d}^{\prime }=O_{d}\times L_{r}$$

A pre-test odd *O_d_* is the ratio of the probability of occurrence of unsatisfactory IAQ to the probability of not having unsatisfactory IAQ, and it is given by

(2) $$O_{d}=\frac{P_{d}}{1-P_{d}}$$

Collective IAQ assessment results are informative in predicting the pre-test probability of having unsatisfactory IAQ. The pre-test probability *P_d_* of unsatisfactory IAQ is calculated by Equation (3), where *N_d_* is the number of unsatisfactory IAQ results in a total of *N* regional assessments.

(3) $$P_{d}=\frac{N_{d}}{N}$$

For the IAQ assessment, an IAQ index θ*_n_* as expressed by Equation (4) is used as a screening test parameter, where λ_j_*—the fractional dose of an assessment parameter with *j* = 1,2, …, *n_j_*—is determined by dividing the exposure level of the *j*-th parameter λ*_j_* by the exposure limit λ_φ,*j*_ over an exposure period. *n*_j_ is the number of parameters measured in the calculation of the IAQ index θ*_n_* [13].

(4) $$\theta_{n}=\frac{1}{n_{j}}\sum_{j=1}^{n_{j}}\lambda_{j}^{*};\;\lambda_{j}^{*}=\frac{\lambda_{j}}{\lambda_{\varphi ,j}}$$

**Figure 1:**
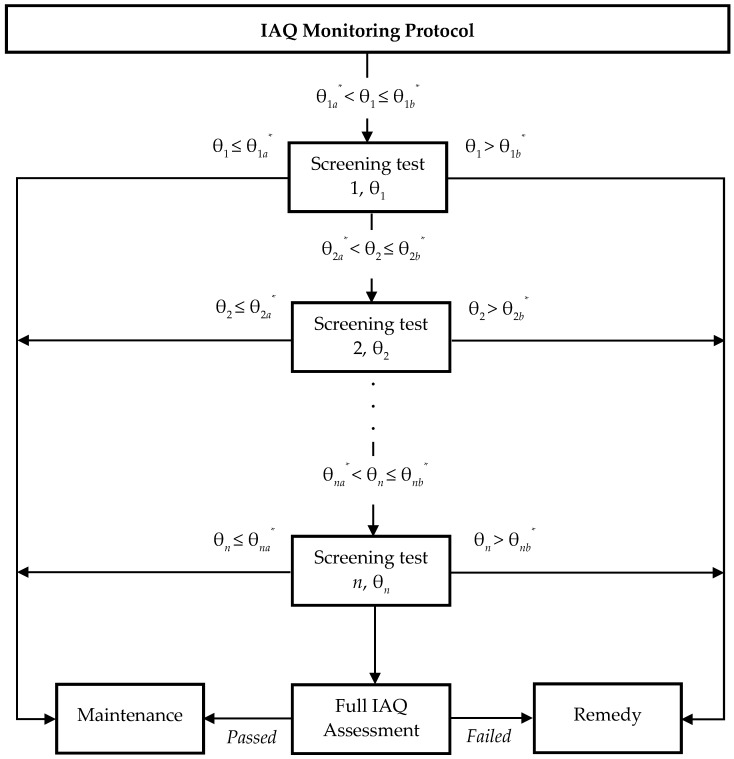
Screening and decision-making process for indoor air quality (IAQ) management.

This index approach uses a stepwise IAQ screening protocol that involves different screening stages where additional IAQ parameters can be included in the index calculation. Figure 1 illustrates the framework of the screening and decision-making process for IAQ management under this approach. The usefulness of each screening step can be objectively assessed by the value of likelihood ratio *L_r_*. A likelihood ratio *L_r_* > 1 indicates a high risk sample having an excessive occurrence of unsatisfactory IAQ, whereas a likelihood ratio *L_r_* < 1 identifies a low risk sample. The likelihood ratio *L_r_* of an IAQ index θ in diagnosing unsatisfactory IAQ can be determined by Equation (5), where *TP* and *TN* are the numbers of test-pass counts and test-fail counts against the screening test parameters θ ≤ θ_a_* and θ > θ_b_*, respectively, *n_TP_* is the total test-pass counts, and *n_TN_* is the total test-fail counts.

(5) $$L_{r}=\frac{TN}{TP}\frac{n_{Tp}}{n_{TN}}$$

### 2.2. IAQ Assessment Database

IAQ Database A contained a total of 525 random samples of Hong Kong air-conditioned open-plan offices (of which 422 were surveyed in 2006 and 103 in 2011) taken from some previous studies [9,14,15]. The offices chosen in this database had similar building materials, style, and age so that the impact of different building factors on the pollutant sources would be minimized. They were individual offices and conference rooms in the size range 10–300 m^2^. Out of them, only 358 met the baseline IAQ testing of all nine IAQ parameters. This database was used to determine the screening levels (i.e., thresholds) of three different IAQ indices (i.e., θ_1_, θ_2_, and θ_3_) for assessing Hong Kong air-conditioned offices based on the likelihood of having unsatisfactory IAQ.

Reported for the first time in this study, IAQ Database B consisted of 2248 Hong Kong air-conditioned open-plan offices randomly collected from various IAQ investigations conducted in the year 2008. These offices were in different building grades and in the size range 10–500 m^2^. They were selected because they covered all regions in Hong Kong and represented the overall IAQ situation in Hong Kong offices. Among them, there were 2002 offices meeting the baseline IAQ testing of all nine IAQ parameters. This database served as a comprehensive dataset for evaluating the feasibility and effectiveness of the screening strategies proposed for preliminary IAQ assessment.

The IAQ sampling method was based on the protocol recommended by the Hong Kong Environmental Protection Department [10]. The arithmetic means (AM), arithmetic standard deviations (SD), and expected failure rate (EFR) of the nine chemical parameters measured against their respective 8-h exposure limits are summarized in Table 1. CO_2_ and TVOC were found to have high failure rates in both Databases A and B. The results for RSP, nitrogen dioxide (NO_2_), formaldehyde (HCHO), TVOC, radon (Rn), and airborne bacteria counts (ABC) in Database A were significantly different from those in Database B (*p* ≤ 0.05, *t*-test). Other parameters, including CO_2_, carbon monoxide (CO), and ozone (O_3_), showed no difference. The office IAQ dissatisfaction rates were 32% and 11% for Databases A and B, respectively. The two independent databases showed no correlation.

### 2.3. IAQ Assessment Database

Likelihood ratios for unsatisfactory IAQ identification using IAQ indices θ_1_, θ_2_, and θ_3_ were compared with the corresponding exposure limits given in the IAQ Certification Scheme. The three indices were categorized into five screening levels based on the testing thresholds (i.e., multilevel likelihood ratios with an order of magnitude *L_r_* = 10 or 0.1) used in a medical test for diagnosing a disease [16]. Except for θ_1_, each category consisted of at least five samples to ensure the fulfilment of statistical requirements. The intermediate levels were distributed evenly for consistency so that comparisons could be made.

Table 2 summarizes the screening results and their corresponding likelihood ratios for IAQ indices θ_1_, θ_2_, and θ_3_. The outcome shows that increasing the number of surrogate parameters incorporated into the index calculation increases sensitivity and specificity of the test, and an IAQ diagnosis using fewer parameters increases uncertainty of the pre-assessment.

**Figure 2:**
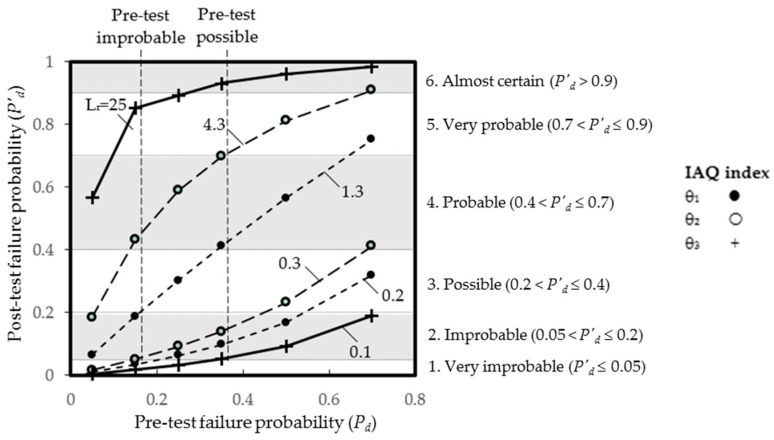
Results of pre- and post-test probabilities (with corresponding verbal probability expressions) under different screening levels. *L_r_*: Likelihood ratio.

The post-test probabilities *P′_d_* of the highest (1.3, 4.3, and 25) and lowest (0.2, 0.3, and 0.1) likelihood ratios for IAQ indices θ_1_, θ_2_, and θ_3_ against the pre-test probabilities *P_d_* from the overall unsatisfactory rates 0.1 to 0.7 for air-conditioned offices are illustrated in Figure 2. As the post-test probability within a range gives the probability of having an unsatisfactory IAQ after screening via the three IAQ indices, it is necessary to set the boundaries for each of the five screening levels using a post-test probability that is significantly high or low in order to rule out most uncertainties. To maximize the unsatisfactory IAQ diagnosed, cut-off values of the IAQ indices should be set with maximum sensitivity, which in turn will lower the specificity of the screening test [17].

For practical uses, verbal probability expressions (VPEs) are used to describe quantitative concepts [18,19]. As depicted in Figure 2, a post-test probability of unsatisfactory IAQ in this study is verbally expressed as: 1, very improbable (≤0.05); 2, improbable (0.05–0.2); 3, possible (0.2–0.4); 4, probable (0.4–0.7); 5, very probable (0.7–0.9); or 6, almost certain (>0.9). At *L_r_* = 25 (i.e., the highest likelihood ratio), θ_3_ is a highly sensitive index to identify unsatisfactory IAQ cases that are from “4, probable” to “6, almost certain”, while θ_1_ is the least sensitive, and θ_2_ can identify most cases of higher than average unsatisfactory IAQ. At *L_r_* = 0.1–0.3 (i.e., the lowest likelihood ratios), any of the three IAQ indices can identify “improbable” cases where the average unsatisfactory rate is up to 0.4. For instance, the screening results of a pre-test “improbable” case (*P′_d_* = 0.15) for θ_1_, θ_2_, and θ_3_ are “2, improbable”, “3, possible”, and “5, very probable”, respectively at *L_r_* = 25, while they are all equal to “1, very improbable” at *L_r_* = 0.1–0.3. An illustration of a pre-test “Possible” case (*P′_d_* = 0.35) is also shown in Figure 2 for comparison.

## 3. Results and Discussion

Table 3 presents the screening results of the 2248 offices in Database B using IAQ indices θ_1_, θ_2_, and θ_3_. Two cases, namely (i) *P_d_* = 0.35 (“3, possible”, representing a higher pre-test failure rate) and (ii) *P_d_* = 0.15 (“2, improbable”, representing a compatible pre-test failure rate) are illustrated as examples. For each screening level, an assessment against all baseline parameters of the IAQ Certification Scheme (i.e., a full test) was performed, and the failure probability *P_j_* was calculated using the true positive counts in *N_j_*, the number of offices screened. In the table, post-test odds *O′_d_* and post-test failure probabilities *P′_d_* are shown along with *N_j_*. While the value of *P_d_* assumed for the screening test was about three times the value given by the full test in case (i), it was compatible with the value given by the full test in case (ii). In general, by assuming one rank higher in the failure probability rankings, the corresponding results would be one rank higher as compared with the full test results. Moreover, when a compatible pre-test probability was assumed, the assessment results of the screening and full tests were similar.

It can be seen that resolution of the screening test using IAQ index θ_1_ is relatively low, as the ranked results involve only two to three out of six VPEs. The results in Table 3a demonstrate that this screening test can identify a small group of samples (183 out of 2248 offices) that are with lower chance of having unsatisfactory IAQ. On the other hand, the ranked results from the screening tests using indices θ_2_ and θ_3_ involve three to four out of six VPEs. The results in Table 3b,c show that θ_2_ and θ_3_ can identify not only the lower risk groups, but also the higher risk ones.

**Figure 3:**
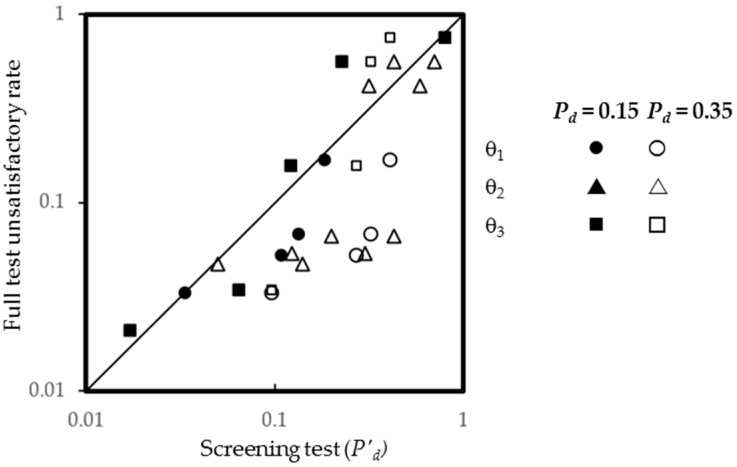
Full test unsatisfactory rate versus post-test failure probability.

Figure 3 plots the full test unsatisfactory rate against the post-test failure probability. It demonstrates that all screening tests give good predictions in general, and the tests using θ_2_ and θ_3_ are good at identifying the high risk groups for unsatisfactory IAQ. Furthermore, it can be seen that a high estimate on the pre-test failure probability results in an overestimate of failure probability.

To exhibit the predictive and problem identification abilities of the step-wise IAQ screening protocol, Database B was screened again consecutively using different IAQ index combinations. The screening results for cases (i) and (ii) are summarized in Table 4; results from the first screening test using θ_1_, θ_2_, and θ_3_ individually are also shown for reference. It is noteworthy that all office conditions were unaltered after each successful screening step in order to maintain statistical consistency. Except for strategy (b) in the intermediate risk group where there is an underestimation, the results once again demonstrate that by assuming one rank higher in the failure probability rankings in case (i), the corresponding results (i.e., *P′_d_*) will be one rank higher as compared with the full test results (i.e., *P_j_*), and by assuming a compatible pre-test probability in case (ii), the assessment results of *P′_d_* and *P_j_* will be similar.

In Table 4, example thresholds *T*_1_ and *T*_2_ represent stringent and lenient IAQ management requirements, respectively. The results demonstrate that all screening strategies (i.e., (a) to (d)) can successfully reduce the number of offices that need a full IAQ test. Overall, the strategies are useful in the probabilistic ranking of having unsatisfactory IAQ, and they have the potential to facilitate cost-effective IAQ management.

## 4. Conclusions

Conducting a full IAQ assessment requires large-scale material and manpower resources. However, an IAQ index can be adopted as a handy screening tool to identify any premises (with poor IAQ) that need more comprehensive IAQ assessments for prioritizing IAQ improvements. This study proposed a stepwise IAQ screening protocol to facilitate cost-effective IAQ management among building owners and managers. The government can also consider a regional IAQ screening using the proposed protocol to diagnose and mitigate IAQ problems in buildings.

## Figures and Tables

**Table 1:** Indoor air quality (IAQ) assessment parameters for air-conditioned offices in Hong Kong.

Parameter	8-h Exposure Limit	Database A AM (SD) [EFR%]	Database B AM (SD) [EFR%]	*p*-Value
CO_2_ (ppm)	1000	658 (151) [7%]	665 (203) [50%]	0.17
CO (µg∙m^−3^)	10,000	1105 (4594) [1%]	1372 (825) [1%]	0.09
RSP (µg∙m^−3^)	180	30 (20) [0%]	27 (30) [3%]	≤0.05
NO_2_ (µg∙m^−3^)	150	27 (17) [0%]	33 (14) [0.4%]	≤0.05
O_3_ (µg∙m^−3^)	120	40 (38) [13%]	40 (19) [3%]	0.39
HCHO (µg∙m^−3^)	100	48 (103) [15%]	29 (22) [13%]	≤0.05
TVOC (µg∙m^−3^)	600	358 (328) [42%]	176 (176) [24%]	≤0.05
Rn (Bq∙m^−3^)	200	46 (39) [0.6%]	68 (41) [6%]	≤0.05
ABC (CFU∙m^−3^)	1000	505 (385) [38.4%]	238 (175) [6%]	≤0.05

AM: arithmetic mean; ABC: airborne bacteria counts; EFR: expected failure rate; HCHO: formaldehyde; Rn: radon; RSP: respirable suspended particulates; SD: standard deviation; TVOC: total volatile organic compounds.

**Table 2:** IAQ indices and likelihood ratios for unsatisfactory IAQ in air-conditioned Hong Kong offices.

k	Screening Level for θ_1_, θ_2_, θ_3_	Unsatisfactory IAQ			Satisfactory IAQ			Likelihood Ratio, *L_r_*		
		Counts (%)			Counts (%)					
		θ_1_	θ_2_	θ_3_	θ_1_	θ_2_	θ_3_	θ_1_	θ_2_	θ_3_
**1**	<0.32	0 (0%)	11 (6.6%)	5 (3%)	0 (0%)	74 (21%)	93 (26%)	/	0.3	0.1
**2**	0.32–0.42	1 (0.6%)	64 (38%)	24 (14%)	10 (2.8%)	165 (46%)	131 (37%)	0.2	0.8	0.4
**3**	0.43–0.53	19 (11%)	61 (37%)	33 (20%)	62 (17%)	96 (27%)	85 (24%)	0.7	1.4	0.8
**4**	0.54–0.64	47 (28%)	23 (14%)	33 (20%)	116 (32%)	19 (5%)	43 (12%)	0.9	2.6	1.7
**5**	≥0.65	99 (59%)	8 (4.8%)	72 (43%)	161 (45%)	4 (1%)	6 (1.7%)	1.3	4.3	25
**Total count**		167 (100%)			358 (100%)					

k is the order of screening level, where k = 1 when θ_n_ < 0.32; k = 2 when 0.32 ≤ θ_n_ ≤ 0.42; k = 3 when 0.43 ≤ θ_n_ ≤ 0.53; k = 4 when 0.54 ≤ θ_n_ ≤ 0.64; and k = 5 when θ_n_ ≥ 0.65.

**Table 3:** Screening levels and assessment results of 2248 offices.

Screening Level	*L_r_*	*N_j_*	(i) Screening Test (*P_d_* = 0.35)			(ii) Screening Test (*P_d_* = 0.15)			Full Test	
			*O*′*_d_*	*P*′*_d_*	Assessment Result	*O*′*_d_*	*P*′*_d_*	Assessment Result	*P_j_*	Assessment Result
**(a) θ_1_**										
0.32–0.42	0.2	183	0.11	0.10	2. Improbable	0.04	0.03	1.Very improbable	0.03	1. Very improbable
0.43–0.53	0.7	444	0.38	0.27	3. Possible	0.12	0.11	2. Improbable	0.05	1. Very improbable
0.54–0.64	0.9	521	0.49	0.33	3. Possible	0.16	0.14	2. Improbable	0.07	2. Improbable
≥0.65	1.3	1100	0.70	0.41	4. Probable	0.23	0.19	2. Improbable	0.17	2. Improbable
**(b) θ_2_**										
<0.32	0.3	510	0.16	0.14	2. Improbable	0.05	0.05	2. Improbable	0.05	1. Very improbable
0.32–0.42	0.8	870	0.43	0.30	3. Possible	0.14	0.12	2. Improbable	0.05	1. Very improbable
0.43–0.53	1.4	570	0.76	0.43	4. Probable	0.25	0.20	3. Possible	0.07	2. Improbable
0.54–0.64	2.6	211	1.40	0.58	4. Probable	0.47	0.32	3. Possible	0.42	4. Probable
≥0.65	4.3	87	2.32	0.70	4. Probable	0.76	0.43	4. Probable	0.56	4. Probable
**(c) θ_3_**										
<0.32	0.1	865	0.05	0.05	1. Very improbable	0.02	0.02	1. Very improbable	0.02	1. Very improbable
0.32–0.42	0.4	819	0.22	0.18	2. Improbable	0.07	0.07	2. Improbable	0.03	1. Very improbable
0.43–0.53	0.8	327	0.43	0.30	3. Possible	0.14	0.12	2. Improbable	0.16	2. Improbable
0.54–0.64	1.7	144	0.92	0.48	4. Probable	0.30	0.23	3. Possible	0.56	4. Probable
≥0.65	25	93	13.5	0.93	6. Almost certain	4.41	0.82	5. Very probable	0.74	5. Very probable

*L_r_*: Likelihood ratio; *N_j_*: true positive counts; *O′_d_*: post-test odds; *P′_d_*: post-test failure probabilities; *P_d_*: pre-test failure probabilities; *P_j_*: full test results.

**Table 4:** IAQ classifications for 2248 offices.

Screening Tests	No. of Offices with Predicted Unsatisfactory IAQ (Unsatisfactory Rate)													
	1. Very Improbable (*P*′*_d_* ≤ 0.05)		2. Improbable (0.05 < *P*′*_d_* ≤ 0.2)		3. Possible (0.2 < *P*′*_d_* ≤ 0.4)		4. Probable (0.4 < *P*′*_d_* ≤ 0.7)		5. Very Probable (0.7 < *P*′*_d_* ≤ 0.9)		6. Almost Certain (*P*′*_d_* > 0.9)		Thresholds *T*_1_ 0.05 < *P*′*_d_* ≤ 0.9	Thresholds *T*_2_ 0.2 < *P*′*_d_* ≤ 0.9
	*N_j_*	*P_j_*	*N_j_*	*P_j_*	*N_j_*	*P_j_*	*N_j_*	*P_j_*	*N_j_*	*P_j_*	*N_j_*	*P_j_*	*N_j_*	*N_j_*
	*P_d_* = 0.35													
θ_1_			183	0.03	965	0.06	1100	0.17					2248	2065
θ_2_			510	0.05	870	0.05	868	0.20					2248	1738
θ_3_	865	0.02	819	0.03	327	0.16	144	0.56			93	0.74	1290	471
(a) θ_1_, θ_2_	126	0.05	435	0.04	872	0.06	741	0.18	74	0.59			2122	1687
(b) θ_1_, θ_3_	737	0.02	448	0.06	837	0.09	133	0.58	3	1	90	0.73	1421	973
(c) θ_2_, θ_3_	852	0.02	407	0.04	630	0.04	190	0.31	80	0.76	89	0.73	1307	900
(d) θ_1_, θ_2_, θ_3_	760	0.03	544	0.03	475	0.04	291	0.21	92	0.73	86	0.72	1402	858
	*P_d_* = 0.15													
θ_1_	183	0.03	2065	0.12									2065	0
θ_2_			1380	0.05	781	0.16	87	0.56					2248	870
θ_3_	865	0.02	1146	0.07	144	0.56			93	0.74			1383	237
(a) θ_1_, θ_2_	546	0.04	937	0.05	682	0.18	83	0.58					1702	765
(b) θ_1_, θ_3_	903	0.02	1119	0.06	133	0.58	3	1	90	0.73			1345	226
(c) θ_2_, θ_3_	945	0.02	968	0.05	166	0.27	80	0.76	35	0.89	54	0.63	1249	281
(d) θ_1_, θ_2_, θ_3_	1007	0.02	806	0.05	255	0.20	91	0.70	35	0.89	54	0.63	1187	381
